# Land-Use Types Regulate Microbial Carbon-Use Efficiency Through Stoichiometric Balance and Resource Limitation in Coastal Saline–Alkaline Soils of the Yellow River Delta

**DOI:** 10.3390/biology15141130

**Published:** 2026-07-11

**Authors:** Haidong Xu, Hongyang Jing, Jianni Sun, Haifei Lu, Rongjia Wang, Qun Gao, Guai Xie, Yiming Wang, Ling Peng

**Affiliations:** 1 Shandong Key Laboratory of Eco-Environmental Science for the Yellow River Delta, Shandong University of Aeronautics, Binzhou 256603, China; 2 Department of Landscape Architecture, Zhejiang Shuren University, Hangzhou 310015, China; 3 College of Forestry and Biotechnology, Zhejiang A&F University, Hangzhou 311300, China; 4 Shandong Society of Soil and Water Conservation, Jinan 250013, China; 5 Jiangxi Academy of Forestry, Nanchang 330032, China

**Keywords:** coastal saline–alkaline soil, land-use types, microbial carbon-use efficiency, ecoenzymatic stoichiometry, microbial resource limitation

## Abstract

Coastal saline–alkaline land has considerable potential for soil carbon sequestration, but how different land-use types affect microbial resource limitation and carbon-use efficiency (CUE) in coastal saline–alkaline soils remains unclear. Four representative land-use types, namely bare land (BL), wetland (WL), grassland (GL), and forest land (FL), were investigated in the coastal saline–alkaline soils of the Yellow River Delta. Soil physicochemical properties, microbial biomass, and extracellular enzyme activities were measured, and ecoenzymatic stoichiometry, microbial resource limitation, and CUE were subsequently calculated. Compared with BL, vegetated land-use types decreased electrical conductivity by 52.1–95.8%, while soil water content, soil nutrient indicators, and microbial biomass indicators increased by 47.1–77.5%, 2.6–136.8%, and 2.2–274.4%, respectively. WL was mainly phosphorus-limited, whereas BL, GL, and FL were primarily nitrogen-limited. Despite relatively high soil organic carbon and nutrient availability, GL showed the strongest N limitation and was the only land-use type showing C limitation. Model-estimated CUE ranged from 0.544 to 0.579 and followed the order FL > BL > WL > GL. Random forest analysis showed that soil physicochemical properties contributed most to CUE variation (42.9%). Structural equation modeling further indicated that soil physicochemical properties were indirectly associated with CUE, mainly through stoichiometric characteristics and microbial resource limitation, showing positive and negative associations, respectively. These findings provide microbial evidence for optimizing land-use patterns, vegetation restoration, and carbon-oriented ecological restoration in coastal saline–alkaline land.

## Simple Summary

Coastal saline–alkaline soils are widely distributed worldwide and have considerable potential for carbon sequestration. However, harsh conditions, such as high salt levels and nutrient limitations, often restrict microbial activity and reduce carbon retention. Different land-use types can improve soil conditions, but their effects on microbial carbon storage processes remain poorly understood. In this study, we compared bare land, wetland, grassland, and forest land in the Yellow River Delta, China. We found that vegetation cover generally reduced soil salinity and increased soil moisture, nutrient availability, and microbial biomass. Microorganisms in different land-use types experienced different nutrient limitations, which influenced how they allocated carbon for growth and nutrient acquisition. Forest land showed the highest microbial carbon-use efficiency, whereas grassland showed the lowest. Soil properties were identified as the main factors associated with microbial carbon retention, largely through their effects on nutrient balance and microbial nutrient demand. These findings improve our understanding of how land-use practices influence soil carbon storage and provide useful guidance for vegetation restoration, sustainable land management, and carbon sequestration in coastal saline–alkaline land.

## 1. Introduction

Coastal saline–alkaline land occurs in transition zones between terrestrial and marine environments and plays an important role in nutrient cycling, biodiversity conservation, and soil organic carbon (SOC) storage [1,2]. However, coastal saline–alkaline soils are commonly subjected to multiple environmental constraints, including high salinity, alkaline conditions, unstable moisture regimes, weak soil structure, and limited organic matter inputs. These constraints can inhibit plant establishment and growth, reduce microbial biomass, suppress extracellular enzyme production, and ultimately disturb the balance between soil carbon inputs and outputs [3,4]. Appropriate land-use practices and vegetation restoration can improve soil environmental conditions by reducing salinity and pH, increasing SOC accumulation and nutrient availability, and stimulating microbial growth and enzyme activities [5,6]. Therefore, understanding how different land-use types affect microbial carbon-use efficiency (CUE) is critical for evaluating the role of land management in improving soil carbon retention and ecological restoration in coastal saline–alkaline land.

Microbial CUE represents the fraction of assimilated carbon that is incorporated into microbial biomass rather than being lost through respiration. As a key indicator linking microbial metabolism with soil carbon cycling, CUE is strongly influenced by ecological stoichiometry and patterns of extracellular enzyme allocation. Soil microorganisms require carbon for energy generation and biomass formation, nitrogen for protein and enzyme synthesis, and phosphorus for nucleic acids, membrane components, and cellular energy transfer. Because the elemental composition of available resources rarely matches microbial nutritional requirements, microorganisms must continuously regulate their metabolic activities to maintain stoichiometric balance. When resource imbalance occurs, microbial communities may increase the production of specific extracellular enzymes to obtain limiting nutrients, alter community composition, or redistribute carbon among growth, respiration, and enzyme synthesis processes [7,8,9]. Accordingly, extracellular enzyme activities provide valuable indicators of microbial nutrient acquisition strategies. β-1,4-glucosidase (BG) is generally regarded as an indicator of carbon acquisition, β-N-acetylglucosaminidase (NAG) and leucine aminopeptidase (LAP) are associated with organic nitrogen acquisition, and alkaline phosphatase (ALP) is linked to phosphorus acquisition. By integrating the relative activities of C-, N-, and P-acquiring enzymes, ecoenzymatic stoichiometry has become a widely adopted framework for evaluating microbial nutrient acquisition strategies [10,11]. Beyond conventional ecoenzymatic stoichiometric approaches, vector analysis and threshold elemental ratio-based models provide additional tools for assessing microbial resource limitation. Vector analysis utilizes enzyme activity ratios to estimate the relative intensity of carbon versus nutrient limitation and nitrogen versus phosphorus limitation [12]. Threshold elemental ratio and vector-threshold models further integrate extracellular enzyme activities, soil resource availability, and microbial stoichiometric characteristics to diagnose microbial metabolic constraints more comprehensively [5]. These approaches are particularly informative because elevated enzyme activity does not necessarily indicate stronger nutrient limitation; it may also reflect greater microbial biomass or increased substrate processing capacity. Recent studies conducted across diverse ecosystems have demonstrated a close relationship between microbial CUE and resource limitation, although the dominant limiting factors differ among climatic regions and ecosystem types [13]. In coastal saline–alkaline soils, microbial CUE may, therefore, be influenced not only by the abundance of SOC, nitrogen, and phosphorus, but also by the balance between resource availability and microbial demand under the combined effects of salinity, moisture variability, and nutrient constraints. Previous investigations have explored the interactions among land use, ecoenzymatic stoichiometry, microbial resource limitation, and CUE in a variety of ecosystems. For instance, multispecies vegetation restoration has been shown to modify ecoenzymatic stoichiometry and microbial nutrient acquisition strategies [4]. Land-use conversion in tropical forests can alter microbial nitrogen limitation and associated nitrogen transformation processes [14]. Similarly, studies conducted in floodplain ecosystems and human-managed landscapes have reported that microbial CUE is regulated through shifts in soil–microbe stoichiometric relationships and resource limitation patterns [6,15]. However, coastal saline–alkaline soils differ substantially from forest, cropland, and floodplain soils because salinity stress, hydrological fluctuations, and vegetation composition can jointly influence substrate diffusion, nutrient accessibility, and microbial metabolic costs. Although the effects of land-use change on soil physicochemical properties have been widely recognized, how different land-use types affect microbial CUE through coordinated changes in resource supply–demand balance and extracellular enzyme allocation remains largely unresolved in coastal saline–alkaline soils.

Accordingly, four typical land-use types within coastal saline–alkaline land, namely bare land, wetland, grassland, and forest land, were selected to examine soil physicochemical properties, microbial biomass and stoichiometry, extracellular enzyme activities, ecoenzymatic stoichiometry, microbial resource limitation, and model-estimated CUE. These four land-use types were selected because they are the dominant land-use patterns on Yellow River Island and are subject to relatively limited human interference and physical soil disturbance compared with agricultural fields or orchards, which undergo tillage and fertilization. The specific objectives were to: (1) determine how land-use types are associated with changes in the saline–alkaline soil microenvironment, including salinity, water content, pH, C, N, and P availability, and soil–microbial stoichiometry; (2) assess how these changes are linked to C-, N-, and P-acquiring enzyme activities, ecoenzymatic stoichiometry, and microbial resource limitation; and (3) identify potential pathways linking soil physicochemical conditions, stoichiometric balance, enzyme allocation, and resource limitation with microbial CUE. We hypothesized that: (1) vegetated land uses ameliorate the saline–alkaline soil microenvironment and increase resource availability compared to bare land; (2) these shifts in soil resources and microbial stoichiometry drive distinct patterns in extracellular enzyme allocation; and (3) microbial CUE depends primarily on the balance between resource supply and microbial demand, with stronger C or N limitation corresponding to lower CUE. These findings will ultimately offer valuable insights into the ecological restoration and sustainable land management of the coastal saline–alkaline region in the Yellow River Delta.

## 2. Materials and Methods

### 2.1. Study Areas and Experimental Design

**Figure 1:**
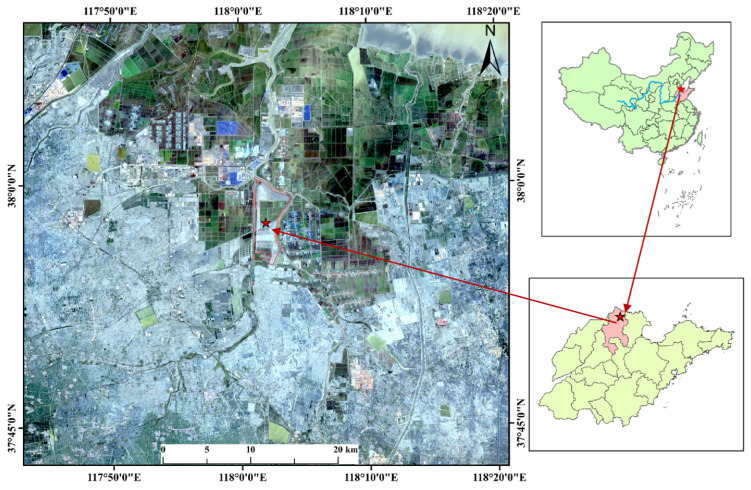
Location of the study area in the Yellow River Delta, China. The red star indicates the sampling site. (The base satellite imagery was obtained from Sentinel-2, and the administrative boundary vector data were obtained from the free public database of the National Geomatics Center of China (NGCC).

This study was conducted on Yellow River Island, Wudi County, Shandong Province, China (118°1′1″–118°4′1″ E, 37°54′57″–38°0′18″ N) (Figure 1). Yellow River Island is situated in the hinterland of the Yellow River Delta, within the transition zone between the coastal plain and tidal flats. The island is bordered by the Bohai Sea to the northeast, the Yellow River to the south, and is surrounded by water. Under the combined effects of fluvial deposition and tidal inundation, the terrain is generally higher in the south and lower in the north, forming a strip-like spatial pattern. The region has a marine-influenced climate, with a mean annual temperature of 13.6 °C; mean annual precipitation of about 600 mm; and annual evaporation of approximately 1960 mm. The soil in the study area is classified as salinized fluvo-aquic soil, which corresponds to Cambisols according to the World Reference Base for Soil Resources 2022 (WRB 2022). It is developed from Yellow River alluvial sediments and has a loamy texture based on the USDA particle-size classification. As a typical saline–alkali area in the Yellow River Delta, Yellow River Island provides a representative setting for examining soil microbial and biochemical processes across different land-use types in coastal saline–alkaline soils [16].

Four representative land-use types were selected by considering both distance from the sea and vegetation cover characteristics: bare land, wetland, grassland, and forest land. These sites differed in vegetation composition, hydrological conditions, and salinity background, which were related to their distance from the sea. Bare land had no vegetation cover and represented saline land awaiting ecological restoration. The wetland was dominated by Suaeda salsa, the grassland by *Setaria viridis* and *Imperata cylindrica*, and the forest land consisted of an artificially planted *Tamarix chinensis* plantation. These land-use types represent typical spatial patterns of coastal saline–alkaline land rather than a strict temporal successional sequence. Consequently, the observed differences among these sites are interpreted as land-use-associated characteristics rather than definitive cause-and-effect relationships, as inherent site-specific baseline conditions (e.g., initial salinity gradients related to distance from the sea) inherently covary with the current vegetation. For each land-use type, four replicate plots (10 m × 10 m each) were established, resulting in 16 plots in total. To strengthen the independence of replicates, adjacent plots were separated by at least 100 m. Before soil collection, surface litter, visible plant residues, and stones were removed from each plot. Surface soil samples from the 0–20 cm layer were then collected using a five-point composite sampling method. Specifically, five randomly distributed soil cores were collected from each plot and mixed thoroughly to form one composite sample. This method was used to obtain a representative plot-level sample and to reduce the influence of small-scale heterogeneity caused by localized root inputs, uneven salt accumulation, and micro-site variation, which are common in coastal saline–alkaline soils. However, because the five soil cores within each plot were composited before analysis, within-plot variation associated with microtopography, rhizosphere effects, and salt patches could not be quantified. Therefore, the 16 composite samples should be interpreted as plot-level representations of each land-use type rather than measurements of fine-scale spatial variability.

Fresh soil samples were transported to the laboratory immediately in an ice box. Upon arrival, residual fine roots, small stones, and other visible debris were carefully removed by hand. The soil was then sieved through a 2-millimeter mesh and separated into several subsamples for further analyses. Fresh soil was used for the determination of soil water content (SWC), while pH, electrical conductivity (EC), soil organic carbon (SOC), soil total nitrogen (STN), soil total phosphorus (STP), and available phosphorus (AP) were analyzed using air-dried soil. Another portion of fresh soil was stored at 4 °C and later used for measuring dissolved organic carbon (DOC), mineral nitrogen (MN, including nitrate nitrogen and ammonium nitrogen), microbial biomass carbon (MBC), microbial biomass nitrogen (MBN), microbial biomass phosphorus (MBP), and extracellular enzyme activities, namely β-glucosidase (BG), β-N-acetylglucosaminidase (NAG), leucine aminopeptidase (LAP), and alkaline phosphatase (ALP).

### 2.2. Soil Physicochemical and Nutrient Analyses

Soil water content (SWC) was determined by the gravimetric method after oven-drying fresh soil at 105 °C until a constant weight was obtained. Soil pH and electrical conductivity (EC) were measured using a pH meter and a conductivity meter, respectively, in a 1:5 (w/v) soil-to-water suspension prepared from air-dried soil samples. Soil organic carbon (SOC) was quantified using the potassium dichromate oxidation procedure. Soil total nitrogen (STN) was measured following Kjeldahl digestion, whereas soil total phosphorus (STP) was determined by the molybdenum–antimony colorimetric method after acid digestion. Dissolved organic carbon (DOC) was extracted from fresh soil using 0.5 mol L^–1^ K_2_SO_4_ and measured with a total organic carbon analyzer. Mineral nitrogen (MN), representing the combined concentration of nitrate nitrogen and ammonium nitrogen, was extracted with 2 mol L^–1^ KCl and analyzed using a continuous-flow analyzer. Available phosphorus (AP) was extracted with 0.5 mol L^–1^ NaHCO_3_ and quantified by molybdenum–antimony colorimetry. All nutrient concentrations were reported on the basis of oven-dry soil mass.

### 2.3. Soil Microbial Biomass and Extracellular Enzyme Activities

Microbial biomass carbon (MBC), microbial biomass nitrogen (MBN), and microbial biomass phosphorus (MBP) were determined using the chloroform fumigation–extraction method [17,18]. In brief, fresh soil samples were divided into fumigated and non-fumigated portions. For MBC and MBN analyses, extraction was carried out using 0.5 mol L^–1^ K_2_SO_4_, whereas 0.5 mol L^–1^ NaHCO_3_ was used for MBP extraction. Microbial biomass values were calculated from the differences between fumigated and non-fumigated extracts, applying extraction conversion factors of 0.45 for MBC, 0.54 for MBN, and 0.40 for MBP.

Extracellular enzyme activities associated with carbon, nitrogen, and phosphorus acquisition were determined using a 96-well microplate fluorometric method [19]. β-glucosidase (BG) was used to represent C-acquiring enzyme activity; β-N-acetylglucosaminidase (NAG) and leucine aminopeptidase (LAP) were used to represent N-acquiring enzyme activities; and alkaline phosphatase (ALP) was used to represent P-acquiring enzyme activity. The fluorogenic substrates were 4-methylumbelliferyl-β-D-glucopyranoside for BG, 4-methylumbelliferyl-N-acetyl-β-D-glucosaminide for NAG, L-leucine-7-amido-4-methylcoumarin for LAP, and 4-methylumbelliferyl phosphate for ALP. Enzyme activities were expressed as nmol substrate released g^–1^ dry soil h^–1^.

### 2.4. Microbial Nutrient Limitation and CUE Calculation

Microbial metabolic limitation was evaluated using the threshold elemental ratio (TER) model and the vector-TER (V-T) model, following previous studies [5,6]. First, the relative enzyme allocation ratios used in the V-T model were calculated as BG/(BG + NAG + LAP) and BG/(BG + ALP), which represented the C:N- and C:P-related enzyme acquisition proportions, respectively. Standardized major axis (SMA) regressions were then conducted between log(BG) and log(NAG + LAP), and between log(BG) and log(ALP), to estimate the normalization constants for N- and P-acquiring enzyme relationships, namely n_0_ and p_0_. The C:N and C:P threshold elemental ratios were calculated as follows:

(1) $${\mathrm{TER}}_{C:N}\;=\;[\mathrm{BG}/(\mathrm{BG}\;+\;\mathrm{NAG}\;+\;\mathrm{LAP})\;\times \;B_{C:N}]\;/\;n_{0}$$

(2) $${\mathrm{TER}}_{C:P}=[\mathrm{BG}/(\mathrm{BG}+\mathrm{ALP})\;\times \;B_{C:P}]\;/\;p_{0}$$

The optimal threshold elemental ratios (TER^0^) were calculated from the maximum nutrient assimilation efficiency (Amax) and maximum microbial carbon-use efficiency (CUE_max_). In this study, Amax_N_ and Amax_P_ were both set to 0.9, and CU_Emax_ was set to 0.6. ${\mathrm{TER}}_{C:N}^{0}$ and ${\mathrm{TER}}_{C:P}^{0}$ were calculated as:

(3) $${\mathrm{TER}}_{C:N}^{0}=\;({\mathrm{Amax}}_{N}\;/\;{\mathrm{CUE}}_{\max})\;\times \;B_{C:N}$$

(4) $${\mathrm{TER}}_{C:P}^{0}=({\mathrm{Amax}}_{P}\;/\;{\mathrm{CUE}}_{\max})\times B_{C:P}$$

Differences between TER and TER^0^, and between soil resource stoichiometry and TER, were calculated as:

(5) $$\Delta {\mathrm{TER}}_{C:N}^{1}\;=\;{\mathrm{TER}}_{C:N}^{0}\;-\;{\mathrm{TER}}_{C:N}\;$$

(6) $$\Delta {\mathrm{TER}}_{C:P}^{1}={\mathrm{TER}}_{C:P}^{0}-{\mathrm{TER}}_{C:P}$$

(7) $$\Delta {\mathrm{TER}}_{C:N}^{2}=L_{C:N}\;-\;{\mathrm{TER}}_{C:N}$$

(8) $$\Delta {\mathrm{TER}}_{C:P}^{2}=L_{C:P}\;-\;{\mathrm{TER}}_{C:P}$$

The TER_EEA_ and TER_L_ indices were further calculated to evaluate relative microbial N versus P limitation:

(9) $${\mathrm{TER}}_{\mathrm{EEA}}\;=\;(\Delta {\mathrm{TER}}_{C:P}^{1}\;/\;B_{C:P})\;-\;(\Delta {\mathrm{TER}}_{C:N}^{1}\;/\;B_{C:N})$$

(10) $${\mathrm{TER}}_{L}=(\Delta {\mathrm{TER}}_{C:P}^{2}\;/\;B_{C:P})-(\Delta {\mathrm{TER}}_{C:N}^{2}\;/{\;B}_{C:N})$$

TER_EEA_ mainly reflects the relative N/P limitation based on extracellular enzyme allocation and microbial biomass stoichiometry, whereas TER_L_ additionally incorporates soil resource stoichiometry. Values greater than zero indicate a stronger relative P limitation, whereas values less than zero indicate a stronger relative N limitation.

Based on the TER framework, the V-T model was further applied to quantify microbial C limitation and relative N/P limitation [5,6]. The vector coordinates were calculated as follows:

(11) x = BG/(BG + ALP)

(12) y=BG/(BG+NAG+LAP)

The reference coordinates x_0_ and y_0_ were derived from standardized major axis (SMA) regressions between x and Δ${\mathrm{TER}}_{C:P}^{1}$, and between y and Δ${\mathrm{TER}}_{C:N}^{1}$, respectively. Microbial C limitation was calculated as the difference between the observed vector length and the reference vector length:

(13) $${\mathrm{VT}}_{C\;\mathrm{limitation}}\;=\;\mathrm{sqrt}(x^{2}\;+\;y^{2})\;-\;\mathrm{sqrt}(x_{0}^{2}\;+\;y_{0}^{2}\;)$$

Relative microbial N/P limitation was calculated as the difference between the observed vector angle and the reference vector angle:

(14) $${\mathrm{VT}}_{N/P\;\mathrm{limitation}}\;=\;\mathrm{degrees}[\mathrm{atan}2(x\;,\;y)]\;-\;\mathrm{degrees}[\mathrm{atan}2(x_{0},\;y_{0})]$$

A VT_C_ limitation value greater than zero indicates a microbial C limitation. A VT_NP_ limitation value greater than zero indicates a stronger relative P limitation, whereas a value less than zero indicates a stronger relative N limitation.

Microbial carbon-use efficiency (CUE) was estimated using a biogeochemical equilibrium model that integrates extracellular enzyme allocation, microbial biomass stoichiometry, and soil resource stoichiometry [6,10]. First, the C:N and C:P stoichiometric constraint terms were calculated as follows:

(15) $$C_{C:N}\;=\;(B_{C:N}\;/S_{C:N})\;\times \;(1\;/\;{\mathrm{EEA}}_{C:N})$$

(16) $$C_{C:P}=\;(B_{C:P}\;/S_{C:P})\times (1\;/\;{\mathrm{EEA}}_{C:P})$$

where B_C:N_ and B_C:P_ are microbial biomass C:N and C:P ratios, calculated as MBC/MBN and MBC/MBP, respectively; S_C:N_ and S_C:P_ are soil resource C:N and C:P ratios, calculated as SOC/STN and SOC/STP, respectively; and EEA_C:N_ and EEA_C:P_ are enzymatic C:N and C:P acquisition ratios, calculated as BG/(NAG + LAP) and BG/ALP, respectively. CUE was calculated as follows:

(17) $$\mathrm{CUE}\;=\;{\mathrm{CUE}}_{\max}\;\times \;\mathrm{sqrt}[(C_{C:N}\;\times \;C_{C:P})\;/\;((K_{C:N}\;+\;C_{C:N})\;\times \;(K_{C:P}\;+\;C_{C:P}))]$$

where CUE_max_ is the maximum microbial carbon-use efficiency and was set to 0.6; and K_C:N_ and K_C:P_ are half-saturation constants for CUE associated with N and P availability, respectively, with both set to 0.5.

### 2.5. Statistical Analyses

All data were examined for normality and homogeneity of variance before statistical analysis, and variables that did not meet these assumptions were log-transformed where needed. One-way analysis of variance (ANOVA) was applied to assess the effects of land-use type on soil physicochemical properties, microbial biomass, extracellular enzyme activities, stoichiometric ratios, microbial resource limitation indices, and CUE. When significant treatment effects were observed, Tukey’s honestly significant difference (HSD) test was used for multiple comparisons, with significance set at *p* < 0.05. Pearson’s correlation analysis was conducted to evaluate the relationships among soil physicochemical properties, microbial biomass, extracellular enzyme activities, stoichiometric ratios, and microbial metabolic limitation indices. Linear regression analysis was further performed to examine the relationships between CUE and microbial metabolic limitation indices, including TER_EEA_, TER_L_, VT_NP_, and VT_C_. Random forest analysis was used to identify the major predictors of CUE and estimate their relative importance.

The significant predictors selected by the random forest model were then incorporated into structural equation modeling (SEM) to evaluate the direct and indirect effects of soil physicochemical properties (pH, EC, MN, STP, STN, and AP), stoichiometry (S_N:P_, EEA_C:N_, EEA_C:P_, and EEA_N:P_), enzyme activity (BG), and resource limitation (VT_NP_ and VT_C_) on CUE. For variable groups with several predictors, principal component analysis (PCA) was performed to reduce the number of variables and limit multicollinearity before SEM analysis. The first PCA axis was retained for each selected group because it accounted for most of the variance (>60% of the total variance; Figure S1), thus representing the main information of each functional category without over-parameterizing the model [20]. The hypothesized SEM pathways were established according to previous studies [6,8,10]. SEM parameters were estimated using Bayesian estimation, and path effects were assessed based on 95% Bayesian credible intervals; this method is suitable for path estimation under small-sample conditions [21]. Model fit was evaluated using χ^2^/df, p-value, comparative fit index (CFI), and goodness-of-fit index (GFI). SEM analyses were performed using AMOS 24.0 (IBM SPSS Inc., Chicago, IL, USA), while all other statistical analyses were carried out in R version 4.4.1 (R Foundation for Statistical Computing, Vienna, Austria).

## 3. Results

### 3.1. Soil Physicochemical Properties, Microbial Biomass, and Stoichiometry Under Different Land-Use Types

Soil physicochemical and biological properties varied significantly among the four land-use types (Table 1). Soil pH was highest in GL and lowest in FL; compared with BL, pH increased by 4.9% in GL but decreased by 3.1% in FL. SWC was significantly higher in WL, GL, and FL than in BL, with the maximum value recorded in WL. EC was highest in BL and decreased by 52.1%, 95.8%, and 95.4% in WL, GL, and FL, respectively. Compared with BL, STN increased by 19.0%, 69.0%, and 40.5% in WL, GL, and FL, respectively, whereas STP increased by 45.0%, 47.5%, and 57.5%, respectively. MN and AP were both highest in GL, showing increases of 136.8% and 42.3% relative to BL, respectively. SOC and DOC were higher in GL and FL than in BL and WL. MBC increased gradually from BL to FL, while MBN and MBP were significantly greater in GL and FL than in BL and WL. S_C:N_ was highest in FL, whereas S_C:P_ and S_N:P_ reached their highest values in GL. B_C:N_ and B_C:P_ were highest in FL, while B_N:P_ showed no significant difference among land-use types.

### 3.2. Soil Extracellular Enzyme Activities and Ecoenzymatic Stoichiometry

**Figure 2:**
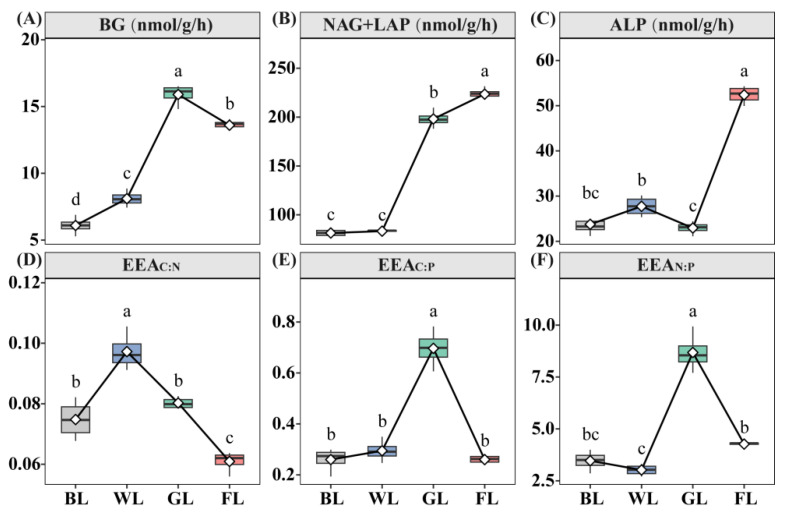
Variation in soil enzyme activities (**A**–**C**) and ecoenzymatic stoichiometric ratios (**D**–**F**) under different land-use types. BL, bare land; WL, wetland; GL, grassland; FL, forest land; EEA_C:N_, the ratio of BG activity to LAP + NAG activity; EEA_C:P_, the ratio of BG activity to ALP activity; EEA_N:P_, the ratio of LAP + NAG activity to ALP activity. Bars indicate the mean ± SE. Different lowercase letters denote significant differences among land-use types, determined by Tukey’s HSD test at *p* < 0.05.

Soil enzyme activities showed significant differences among land-use types (Figure 2). BG activity followed the order GL > FL > WL > BL, increasing by 33.0%, 161.1%, and 123.3% in WL, GL, and FL, respectively, compared with BL. NAG + LAP activity did not differ significantly between BL and WL, but it was significantly higher in GL and FL; compared with BL, it increased by 143.4% and 174.5% in GL and FL, respectively. ALP activity was highest in FL, with an increase of 120.5% compared with BL, whereas GL showed the lowest ALP activity among the vegetation-covered land-use types. For ecoenzymatic stoichiometry, EEA_C:N_ was highest in WL and lowest in FL, while EEA_C:P_ and EEA_N:P_ were highest in GL. EEA_C:P_ showed no significant difference among BL, WL, and FL, whereas EEA_N:P_ was lowest in WL.

### 3.3. Microbial Carbon and Nitrogen/Phosphorus Limitations

**Figure 3:**
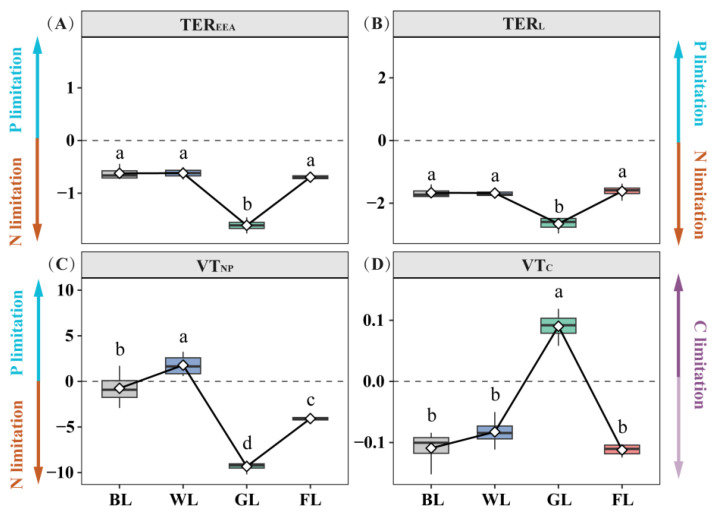
Microbial resource limitation across different land-use types: (**A**) TER_EEA_; (**B**) TER_L_; (**C**) VT_C_; (**D**) VT_NP_. BL, bare land; WL, wetland; GL, grassland; FL, forest land. Bars indicate the mean ± SE. Different lowercase letters denote significant differences among land-use types, determined by Tukey’s HSD test at *p* < 0.05.

According to the TER_EEA_ and TER_L_ models, all land-use types were characterized by N limitation, with the strongest limitation observed in GL (Figure 3). Relative to BL, N limitation based on TER_EEA_ increased by 159.7% in GL and 12.9% in FL, whereas WL remained similar to BL. Based on TER_L_, N limitation in GL increased by 59.3% compared with BL, while WL and FL showed values comparable to those of BL. The VT_NP_ model distinguished the patterns of N and P limitation among land-use types: WL was P-limited, whereas BL, GL, and FL were N-limited, with the strongest N limitation occurring in GL, followed by FL and BL. The VT_C_ model indicated that C limitation occurred only in GL.

### 3.4. Correlations Between Microbial Resource Limitation and Soil Properties

**Figure 4:**
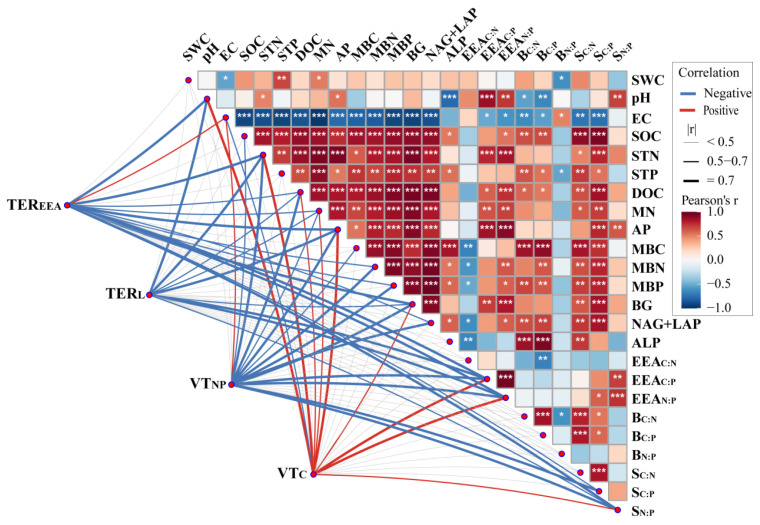
Relationships between microbial resource limitation indices and soil physicochemical properties, microbial biomass, enzyme activities, and stoichiometric ratios. Red, blue, and gray lines indicate significant positive correlations, significant negative correlations, and non-significant correlations, respectively. Red and blue squares represent positive and negative correlations, respectively. Asterisks indicate significance levels (* *p* < 0.05, ** *p* < 0.01, *** *p* < 0.001).

Correlation analysis showed that microbial resource limitation indices were strongly associated with soil nutrient conditions, enzyme activities, microbial biomass, and stoichiometric ratios (Figure 4). TER_EEA_ and TER_L_ presented generally similar correlation patterns and were mainly negatively associated with nutrient-related variables and enzyme stoichiometric ratios, including pH, AP, STN, MN, DOC, BG, S_N:P_, EEA_C:P_, and EEA_N:P_. VT_NP_ was negatively related to most nutrient-, microbial biomass-, and enzyme-related variables, including SOC, STN, DOC, MN, AP, MBC, MBN, MBP, BG, NAG + LAP, S_C:P_, S_N:P_, EEA_C:P_, and EEA_N:P_, but showed a positive correlation with EC. By contrast, VT_C_ was positively correlated with nutrient availability and enzyme stoichiometric ratios, especially pH, STN, MN, AP, DOC, BG, S_N:P_, EEA_C:P_, and EEA_N:P_, but was negatively correlated with EC. Among enzyme-related variables, BG and NAG + LAP exhibited a similar correlation pattern, with both being positively associated with soil C and nutrient variables, microbial biomass, and soil stoichiometric ratios, but negatively associated with EC. ALP was positively related to microbial biomass and stoichiometric ratios, particularly MBC, B_C:N_, B_C:P_, and S_C:N_, whereas negative correlations were observed with pH and EEA_C:N_. EEA_C:P_ and EEA_N:P_ showed similar positive associations with nutrient availability and S_N:P_, while EEA_C:N_ was negatively correlated with microbial biomass and enzyme activities.

### 3.5. Microbial Carbon-Use Efficiency

**Figure 5:**
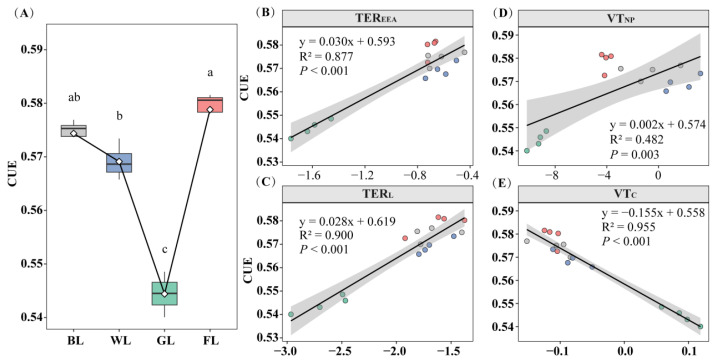
Variation in microbial carbon-use efficiency (CUE) among different land-use types and its relationships with microbial resource limitation models: (**A**) CUE under different land-use types; regression relationships between CUE and (**B**) TER_EEA_, (**C**) TER_L_, (**D**) VT_NP_, and (**E**) VT_C_. BL, bare land; WL, wetland; GL, grassland; FL, forest land. Different lowercase letters denote significant differences among land-use types, determined by Tukey’s HSD test at *p* < 0.05.

Microbial carbon-use efficiency (CUE) varied significantly among land-use types (Figure 5A), with the order FL > BL ≈ WL > GL. Compared with GL, CUE increased by about 5.6% in BL and WL and by 7.4% in FL. Relative to BL, CUE was reduced by about 5.3% in GL, whereas FL showed a small increase. Linear regression analysis indicated significant positive relationships of CUE with TER_EEA_ (R^2^ = 0.877, *p* < 0.001), TER_L_ (R^2^ = 0.900, *p* < 0.001), and VT_NP_ (R^2^ = 0.482, *p* = 0.003) (Figure 5B–D). In contrast, CUE was negatively associated with VT_C_ (R^2^ = 0.955, *p* < 0.001) (Figure 5E).

### 3.6. Key Predictors and Pathways Regulating CUE

**Figure 6:**
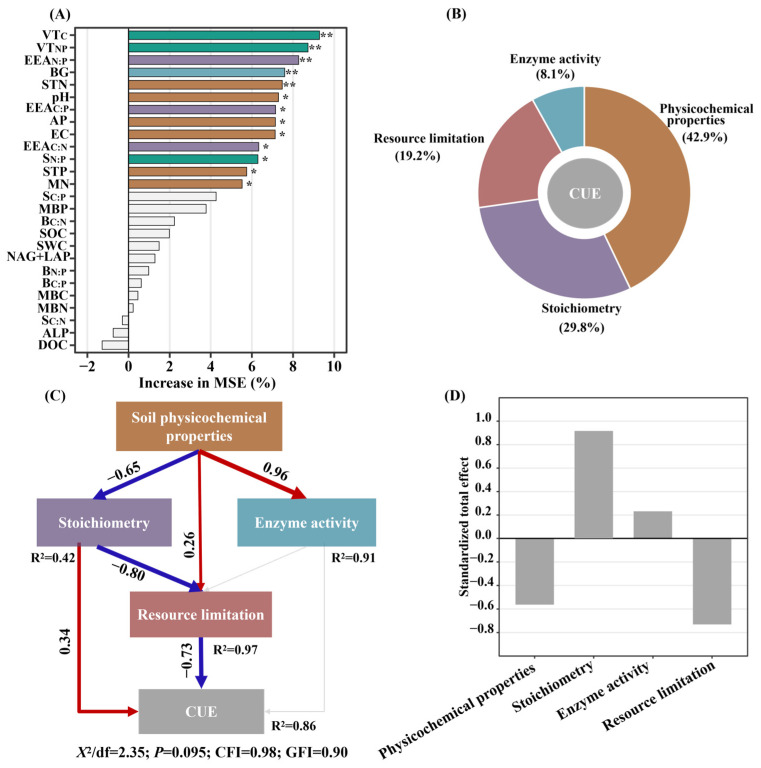
Random forest analysis of microbial carbon-use efficiency (CUE) under different land-use types: (**A**) significant predictors of CUE; (**B**) relative contributions of predictors to CUE; (**C**) direct and indirect effects of predictors on CUE based on the structural equation model; (**D**) standardized total effects of predictors on CUE. Asterisks indicate significance levels (* *p* < 0.05, ** *p* < 0.01).

Random forest analysis was initially used to identify the main predictors of CUE across different land-use types, followed by structural equation modeling (SEM) to assess their direct and indirect pathways (Figure 5). The random forest model selected VT_C_, VT_NP_, EEA_N:P_, BG, STN, pH, EEA_C:P_, AP, EC, EEA_C:N_, S_N:P_, STP, and MN as important predictors of CUE (Figure 6A). After these predictors were grouped into functional categories, soil physicochemical properties made the largest contribution to CUE variation (42.9%), followed by stoichiometry (29.9%), resource limitation (19.2%), and enzyme activity (8.1%) (Figure 6B). SEM further distinguished the direct and indirect effects of these predictor groups on CUE (Figure 6C). Stoichiometry showed the strongest positive total effect on CUE (0.918), including both a direct effect (0.336) and an indirect effect (0.582). Enzyme activity had a positive direct effect on CUE (0.232). Conversely, resource limitation showed a strong negative direct effect on CUE (–0.732), while soil physicochemical properties showed a negative total effect on CUE (–0.563) through indirect pathways. Overall, stoichiometry had the strongest total effect on CUE, whereas resource limitation and soil physicochemical properties showed negative total effects (Figure 6D).

## 4. Discussion

### 4.1. Effects of Land-Use Type on Soil Physicochemical Properties, Microbial Biomass, and Stoichiometry

The composition and management of different land-use types strongly influence the soil microenvironment in coastal saline–alkaline ecosystems. In this study, land-use type significantly affected soil EC, SWC, pH, C, N, and P availability, as well as microbial biomass and stoichiometric characteristics. These results support the first hypothesis, indicating that vegetation cover can improve soil microhabitat conditions and create a more favorable environment for microbial growth. This finding is broadly consistent with previous studies on coastal reclamation and saline soil restoration [1,22]. Compared with bare land, wetland, grassland, and forest land had lower EC but higher SWC, SOC, DOC, nutrient availability, and microbial biomass, suggesting that vegetation cover reduced the environmental constraints associated with high salinity, limited organic inputs, and weak microbial activity in unvegetated saline–alkaline soils. In bare land, the absence of vegetation cover and root inputs likely promoted surface salt accumulation under strong evaporation. High salinity may further reduce soil water availability, increase osmotic stress, and limit substrate diffusion and microbial activity. Thus, the coexistence of higher EC and lower MBC, MBN, and MBP in bare land reflects the combined limitations imposed by salt stress and inadequate substrate supply on microbial habitats.

Grassland and forest land showed clear differences in the pathways by which soil resources were improved, providing additional evidence for functional variation among vegetation types. Overall, grassland and forest land appeared to represent two contrasting resource-regulation patterns: a nutrient-availability pattern, characterized by higher AP and MN contents in grassland, and a microbial carbon-accumulation pattern, characterized by higher SOC and microbial biomass in forest land. These differences were shown not only as changes in single nutrient concentrations but also in the regulation of soil and microbial stoichiometry [23]. Grassland was mainly composed of herbaceous species, such as *Setaria viridis* and *Imperata cylindrica*, which generally have shallow and quickly renewing root systems, fast aboveground residue turnover, and active rhizosphere exudation. These characteristics likely supported the accumulation of MN, AP, and STN in surface soil [24,25]. However, such active rhizosphere processes may also have led to asynchronous elemental accumulation, causing higher S_C:P_ and S_N:P_ in grassland and a certain level of resource stoichiometric imbalance. In contrast, woody vegetation, such as the *Tamarix chinensis* forest, relies on relatively stable litter inputs, continuous root-derived C supply, and long-term maintenance of vegetation structure, thereby promoting SOC accumulation, STP enrichment, and MBC formation. These processes further increased the relative accumulation of microbial biomass C over N and P, as shown by the higher B_C:N_ and B_C:P_ in forest land [26]. These findings suggest that, in coastal saline–alkaline environments, land-use type not only controls the absolute supply of individual nutrients but also forms distinct microbial habitats and nutrient-use patterns by regulating coupled changes in soil resource structure and microbial biomass elemental composition.

### 4.2. Effects of Land-Use Type on Soil Extracellular Enzyme Activities and Ecoenzymatic Stoichiometry

Changes in land-use type can reshape microbial functional activity by altering enzyme-mediated nutrient acquisition. The results showed that the activities of C-, N-, and P-acquiring extracellular enzymes and their ecoenzymatic stoichiometric ratios differed significantly among land-use types. This pattern supports the second hypothesis, suggesting that differences in soil resource availability and microbial biomass under different land-use types can drive adaptive allocation of extracellular enzymes. Specifically, C- and N-acquiring enzymes involved in organic carbon and nitrogen degradation, including BG, NAG, and LAP, were significantly higher in grassland and forest land than in bare land and wetland. Correlation analysis further confirmed that C- and N-acquiring enzyme activities were positively associated with organic carbon (SOC and DOC), labile nutrients (STN and MN), and microbial biomass, but negatively correlated with soil EC. This suggests that the enhancement of enzyme activity under vegetation cover was not only a result of increased substrate inputs, such as plant litter and root exudates, but also reflected the combined effects of greater substrate availability and reduced salinity stress. Together, these changes improved microbial habitat conditions and enzymatic reaction efficiency, thereby increasing community-level enzyme production [22,27]. These findings extend this understanding to coastal saline–alkaline soils and show that decreased salinity, greater microbial biomass, and enhanced vegetation inputs jointly regulate extracellular enzyme activity.

However, the response of P-acquiring enzymes, particularly ALP, differed from that of C- and N-acquiring enzymes, further emphasizing the distinct signaling role of ecoenzymatic stoichiometry in microbial resource acquisition strategies. Although AP was highest in grassland soils, ALP activity remained relatively low; conversely, forest soils exhibited the highest ALP activity. This contrast indicates that P-acquiring enzyme production does not simply increase with absolute soil nutrient concentrations but is also regulated by microbial P demand, biomass accumulation, and stoichiometric status. Elevated AP in grassland may have reduced the relative need for phosphatase investment, causing microorganisms to preferentially allocate resources toward C- and N-acquiring processes, resulting in the highest ecoenzymatic ratios (EEA_C:P_ and EEA_N:P_). In forest soils, higher microbial biomass carbon and increased microbial C:N and C:P ratios (i.e., MBC, B_C:N_, and B_C:P_) suggest potential P limitation under strong C accumulation. To adjust to this stoichiometric imbalance, microorganisms may have upregulated P-acquiring enzyme synthesis, thereby substantially reducing EEA_C:P_ and EEA_N:P_ in forest soils [9]. This mechanism indicates that different vegetation types, by modifying resource input quality and microbial biomass stoichiometry, can drive adaptive changes in microbial nutrient-use strategies and extracellular enzyme allocation [28,29].

### 4.3. Effects of Land-Use Type on Microbial Resource Limitation

Microbial resource limitation varied among land-use types, indicating that shifts in vegetation cover and soil conditions can alter microbial C, N, and P acquisition strategies in coastal saline–alkaline soils. The VT_NP_ model indicated that wetland was mainly P-limited, whereas bare land, grassland, and forest land were primarily N-limited. This broader tendency toward N limitation was also supported by the TER_EEA_ and TER_L_ models, suggesting that microbial demand for N was generally stronger than that for P in most land-use types. These findings are consistent with previous studies showing that land-use change can modify microbial nutrient limitation by altering microbial biomass, soil nutrient availability, and N transformation processes [6,14]. However, the P limitation observed in wetland may be linked to its distinct hydrological conditions and halophytic vegetation. Wetland had the highest soil water content among the four land-use types, and prolonged or repeated water saturation may alter soil redox conditions, thereby influencing P sorption–desorption, mineral transformation, and nutrient diffusion. Under such conditions, microbial access to soil P may not be fully represented by bulk nutrient concentrations. In addition, the dominance of halophytic vegetation, such as *Suaeda salsa*, may regulate rhizosphere nutrient cycling under the combined effects of salinity and water stress. These hydrological and vegetation-related factors may partly explain why wetland differed from the other land-use types in microbial N/P limitation patterns [30,31].

Further comparison of the VT_NP_ and VT_C_ models showed that grassland exhibited the strongest N limitation and was the only land-use type with apparent C limitation, despite its relatively high STN, MN, AP, and SOC contents. This finding supports the third hypothesis, indicating that microbial resource limitation is controlled not only by the absolute size of soil nutrient pools, but also by the balance between resource supply and microbial demand [7]. The coexistence of high BG activity and apparent C limitation in grassland is not contradictory. High BG activity suggests that microorganisms increased enzymatic investment in C acquisition, which is consistent with stronger C demand. However, the relatively high SOC content in grassland does not necessarily indicate a sufficient supply of microbial-accessible C. Part of the SOC pool may occur in relatively recalcitrant forms or be physically protected within soil aggregates, thereby reducing its immediate availability to microorganisms [32]. In addition, the relatively high N availability in grassland may stimulate microbial biomass accumulation and metabolic activity, further increasing microbial demand for C and leading to stoichiometric imbalance [33]. Similarly, elevated NAG and LAP activities suggest enhanced microbial investment in N acquisition, indicating that microbial N demand may also increase under active nutrient cycling. Therefore, the strong C and N limitation in grassland likely reflects a mismatch between effective resource availability and microbial metabolic demand, rather than a simple deficiency of total soil C or nutrients. Future studies should further distinguish labile and recalcitrant C fractions and incorporate microbial community and functional analyses to better clarify the mechanisms underlying microbial resource limitation in coastal saline–alkaline grasslands [34,35].

### 4.4. Regulatory Mechanisms of Different Land-Use Types on CUE

Microbial carbon-use efficiency (CUE) varied clearly among land-use types, with the greatest values found in forest land and the lowest values recorded in grassland. This pattern partly supports the third hypothesis, showing that microbial CUE is not controlled only by nutrient availability but is more strongly linked with the balance between resource supply and microbial metabolic needs [7]. In this study, grassland soils contained relatively high STN, MN, and AP levels, together with strong C- and N-acquiring enzyme activities; however, the lowest CUE was still observed. This difference indicates that high nutrient availability does not always result in higher microbial carbon retention when microbial resource demand and metabolic investment are also increased. The reduced CUE in grassland may be associated with stronger resource constraints, especially the simultaneous occurrence of C and N limitation [36]. Regression analysis showed that CUE was positively related to N/P limitation indices (TER_EEA_, TER_L_, and VT_NP_) but negatively related to VT_C_, which reflects microbial C limitation. Together with the resource limitation results, these associations suggest that stronger C limitation was linked with lower microbial CUE in grassland. Under these conditions, a greater proportion of assimilated carbon may be allocated by microorganisms to extracellular enzyme production, substrate acquisition, and maintenance metabolism rather than to biomass formation [37]. This explanation agrees with the view that microbial CUE is controlled by the stoichiometric matching between resource supply and microbial demand [7,8]. In contrast, higher CUE was observed in forest land, which may be related to its larger MBC pool, greater SOC availability, and stronger investment in P-acquiring enzymes. These features probably weakened C limitation and promoted microbial biomass accumulation, thereby supporting more efficient microbial carbon retention.

The combined random forest and SEM analyses further explained the regulatory pathways of CUE. Random forest analysis identified soil physicochemical properties as the main apparent contributor to CUE variation, suggesting that salinity, pH, moisture, and nutrient availability form the environmental background for microbial carbon allocation. However, SEM showed that stoichiometric characteristics had the strongest positive total effect on CUE, whereas resource limitation had the strongest negative direct effect. This difference between variable importance and pathway effects indicates that the effects of land use on CUE are likely transferred through changes in soil and microbial stoichiometry, enzyme allocation, and resource limitation, rather than through soil physicochemical properties alone. In the coastal saline–alkaline soils of the Yellow River Delta, forest land appeared to improve microbial carbon retention by supporting higher microbial biomass and a more suitable resource balance [38]. By comparison, grassland had lower CUE despite higher nutrient availability, probably due to stronger microbial C and N demand. Therefore, microbial CUE in this system should be understood as a result of resource supply–demand matching and metabolic allocation, rather than as a simple response to nutrient enrichment [39]. Furthermore, it is important to note that our findings are based on a spatial comparative approach. While strong associations were observed between land-use types, soil microenvironments, and microbial CUE, these relationships remain correlative. Future temporal or manipulative experiments are necessary to disentangle the direct causal effects of vegetation from the pre-existing site-specific characteristics.

### 4.5. Ecological Implications and Research Limitations

The results of this study suggest that the ecological assessment of coastal saline–alkaline land restoration should go beyond common indicators such as vegetation cover, reduced soil salinity, or nutrient enrichment. Although these indicators reflect improvements in habitat quality, they do not fully explain how microorganisms allocate carbon under different resource conditions. Microbial resource limitation and CUE provide additional functional information because they indicate whether improved soil conditions are associated with more efficient microbial carbon use and potential carbon retention. Therefore, incorporating microbial metabolic indicators into restoration assessment may help distinguish land-use types that mainly increase nutrient availability from those that more effectively support microbial carbon stabilization.

From a soil-process perspective, the observed differences among land-use types may reflect several surface soil processes, including salinization or desalinization, organic matter accumulation, nutrient transformation, soil–microbial stoichiometric adjustment, and enzyme-mediated C, N, and P cycling. These processes are particularly important in coastal saline–alkaline soils, where strong evaporation, shallow groundwater, tidal influence, and vegetation development jointly affect salt accumulation, substrate availability, microbial activity, and carbon turnover. From a management viewpoint, land-use types with high nutrient availability may not necessarily support high microbial CUE if microbial C or N demand also increases. In contrast, vegetation types that increase microbial biomass while maintaining a more balanced resource environment may be more beneficial for soil carbon retention. These findings suggest that vegetation selection and land-use planning in coastal saline–alkaline land should aim not only to reduce salinity and improve soil fertility but also to optimize soil–microbial stoichiometric balance and alleviate excessive microbial resource limitation.

Several limitations should be noted when interpreting these findings. First, the four land-use types represent current spatial differences in vegetation and surface soil conditions on Yellow River Island rather than a strict temporal restoration sequence; therefore, the observed differences should be interpreted mainly as land-use-associated patterns. Second, this study focused on 0–20 cm surface soils and did not examine vertical profile-scale processes, such as salt redistribution along the soil profile, capillary salt movement driven by groundwater, diagnostic horizon development, or salic/natric horizon formation. Third, soluble salt composition, soil solution chemistry, cation exchange capacity, exchangeable sodium percentage, sodium adsorption ratio, and the ratio of coarse fragments to fine earth were not measured; therefore, the detailed ionic composition and geochemical mechanisms of salinization could not be fully evaluated. Fourth, the relatively small sample size may reduce the statistical robustness of the path analysis, and the SEM results should be considered exploratory evidence for potential pathways rather than definitive causal proof. Finally, the reported CUE and resource limitations were estimated using ecoenzymatic stoichiometric models. While this approach efficiently assesses community-level metabolic potentials across spatial scales, its reliance on equilibrium assumptions precludes capturing real-time carbon fluxes. In contrast, direct isotopic tracing offers accurate empirical measurements of actual microbial growth and respiration, though typically as labor-intensive, short-term snapshots [40]. Consequently, our discussions of microbial carbon retention reflect theoretical stoichiometric allocations rather than directly measured fluxes. Future research should integrate direct isotope-based measurements with microbial multi-omics and long-term monitoring to further elucidate carbon allocation in saline–alkaline soils.

## 5. Conclusions

In summary, this study demonstrates that different land-use types strongly regulate CUE in coastal saline–alkaline soils through coordinated changes in soil physicochemical properties, microbial biomass stoichiometry, extracellular enzyme allocation, and overall resource limitation. Grassland and forest land showed contrasting microbial carbon allocation strategies, reflecting the complex interaction between environmental resource inputs and microbial metabolic demands. Specifically, grassland microorganisms experienced strong combined C and N limitation despite high nutrient availability, which may have shifted assimilated carbon toward enzyme production and maintenance respiration rather than biomass accumulation. By contrast, forest land microorganisms benefited from higher microbial biomass and a more balanced nutrient supply, which likely alleviated C limitation and improved CUE. Together, these findings provide a mechanistic framework for understanding microbial carbon dynamics under different land-use types and identify practical pathways for optimizing carbon sequestration in saline–alkaline environments.

## Figures and Tables

**Table 1:** Chemical and biological properties of soils under different land-use types. Data are presented as mean ± SE. Different lowercase letters indicate significant differences among land-use types, as determined by Tukey’s HSD test at *p* < 0.05.

Variables	BL	WL	GL	FL
pH	8.4 ± 0.02 b	8.45 ± 0.01 b	8.81 ± 0.01 a	8.14 ± 0.03 c
SWC (%)	16.07 ± 1.93 b	28.52 ± 2.72 a	23.64 ± 1.04 a	24.91 ± 0.46 a
EC (mS/cm)	41.39 ± 2.01 a	19.82 ± 0.16 b	1.729 ± 0.03 c	1.884 ± 0.12 c
SOC (g/kg)	2.66 ± 0.11 b	3.9 ± 0.05 b	5.89 ± 0.07 a	6.22 ± 0.63 a
STN (g/kg)	0.42 ± 0.01 d	0.5 ± 0.01 c	0.71 ± 0.01 a	0.59 ± 0.01 b
STP (g/kg)	0.4 ± 0.01 c	0.58 ± 0.01 b	0.59 ± 0.01 b	0.63 ± 0.01 a
DOC (mg/kg)	26.96 ± 0.82 b	28.15 ± 1.15 b	51.14 ± 2.07 a	48.59 ± 2.29 a
MN (mg/kg)	4.73 ± 0.05 d	8.11 ± 0.12 c	11.2 ± 0.22 a	9.83 ± 0.14 b
AP (mg/kg)	12.44 ± 0.28 c	12.76 ± 0.23 c	17.7 ± 0.13 a	14.8 ± 0.5 b
MBC (mg/kg)	54.56 ± 2.47 d	68.74 ± 1.49 c	127.1 ± 0.98 b	204.28 ± 5.37 a
MBN (mg/kg)	11.69 ± 0.64 b	11.95 ± 0.41 b	22.43 ± 0.84 a	23.52 ± 1.71 a
MBP (mg/kg)	2.46 ± 0.13 b	2.99 ± 0.11 b	5.4 ± 0.03 a	5.87 ± 0.2 a
B_C:N_	4.74 ± 0.49 b	5.76 ± 0.08 b	5.69 ± 0.18 b	8.81 ± 0.61 a
B_C:P_	22.24 ± 0.33 b	23.02 ± 0.41 b	23.53 ± 0.18 b	34.98 ± 1.99 a
B_N:P_	4.83 ± 0.46 a	4 ± 0.02 a	4.15 ± 0.15 a	4.02 ± 0.32 a
S_C:N_	6.27 ± 0.25 b	7.77 ± 0.19 b	8.32 ± 0.1 ab	10.5 ± 1.01 a
S_C:P_	6.69 ± 0.27 b	6.73 ± 0.13 b	9.96 ± 0.15 a	9.82 ± 1.09 a
S_N:P_	1.07 ± 0.01 b	0.87 ± 0.02 c	1.2 ± 0.03 a	0.93 ± 0.02 c

Note: BL, bare land; WL, wetland; GL, grassland; FL, forest land; SWC, soil water content; EC, electrical conductivity; SOC, soil organic carbon; STN, soil total nitrogen; STP, soil total phosphorus; DOC, dissolved organic carbon; MN, mineral nitrogen, the sum of NH_4_^+^-N and NO_3_^−^-N; AP, available phosphorus; MBC, microbial biomass carbon; MBN, microbial biomass nitrogen; MBP, microbial biomass phosphorus; B_C:N_, the ratio of MBC to MBN; B_C:P_, the ratio of MBC to MBP; B_N:P_, the ratio of MBN to MBP; S_C:N_, the ratio of SOC to STN; S_C:P_, the ratio of SOC to STP; S_N:P_, the ratio of STN to STP.

## Data Availability

The original contributions presented in this study are included in the article/Supplementary Materials. Further inquiries can be directed to the corresponding author.

## Supplementary Materials

The following supporting information can be downloaded at: https://www.mdpi.com/article/10.3390/biology15141130/s1, Figure S1: Principal component analysis (PCA) of the grouped predictor variables used in the structural equation modeling (SEM) analysis.
