# Supplementary material for: Land-Use Types Regulate Microbial Carbon-Use Efficiency Through Stoichiometric Balance and Resource Limitation in Coastal Saline–Alkaline Soils of the Yellow River Delta
Source: Biology (Basel). 2026 Jul 11;15(14):1130. doi: 10.3390/biology15141130 (PMC13404933; doi:10.3390/biology15141130)
Supplement: Supplementary file 1 [file biology-15-01130-s001.zip › biology-4423713-supplementary.pdf]

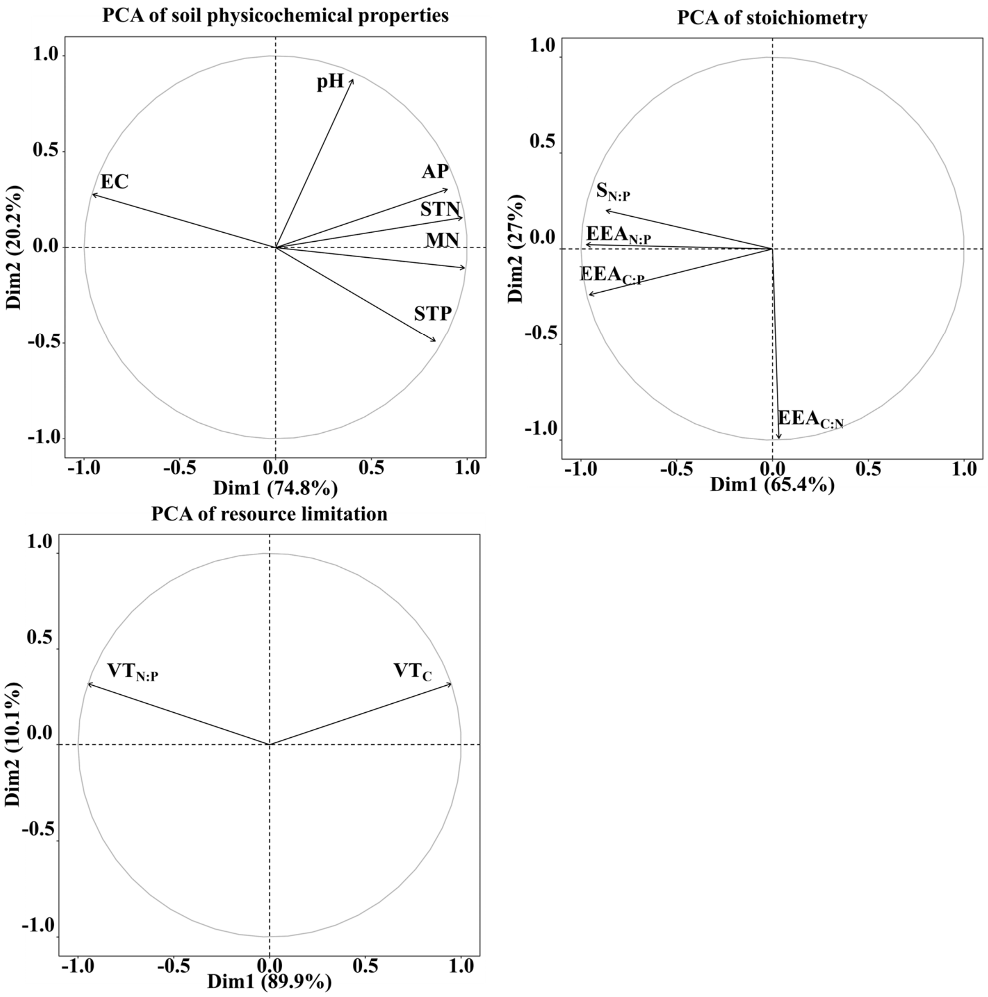

Figure S1. Principal component analysis (PCA) of the grouped predictor variables used in the structural equation modeling (SEM) analysis.
